# POPULATION REFERENCE VALUES FOR PEAK EXPIRATORY FLOW IN OLDER US ADULTS

**DOI:** 10.1093/geroni/igad104.2788

**Published:** 2023-12-21

**Authors:** Patrick Donahue, Aparna Balasubramanian, Anis Davoudi, Jennifer Schrack, Amal Wanigatunga, Michelle Carlson

**Affiliations:** Johns Hopkins University, Baltimore, Maryland, United States; Johns Hopkins University, Baltimore, Maryland, United States; Johns Hopkins University, Baltimore, Maryland, United States; Johns Hopkins University, Baltimore, Maryland, United States; Johns Hopkins School of Public Health, Baltimore, Maryland, United States; Johns Hopkins Bloomberg School of Public Health, Baltimore, Maryland, United States

## Abstract

Respiratory function predicts physical and cognitive health outcomes in older adults. Peak expiratory flow (PEF) is simple, inexpensive, and has been validated against traditional respiratory function measures (FEV1, FVC). PEF has additional utility because it measures maximal respiratory effort without relying on spirometry equipment. However, there is a lack of epidemiological data to support PEF reference values for aging populations, especially those ≥80 years. The National Health and Aging Trends Study (NHATS) is a large, nationally representative sample of US adults ages ≥65 that offers a unique opportunity to develop PEF population reference values. Using a healthy subsample from NHATS Round 1 (N=2,082), excluding those with history of smoking, respiratory, or cardiovascular diseases, we applied NHATS sampling weights to estimate age- and sex-specific PEF percentiles. The sample was stratified by sex (68.9% female) and categorized into six, 5-year age groups: 65-69, 70-74, … ≥90 (36.6% age ≥80 years). In a secondary analysis, we estimated age- and sex-specific PEF/height3 percentiles, per current recommendations. We calculated 5th, 95th, and 10th, 20th, … 90th percentiles, by age and sex, for both absolute and height-adjusted PEF. PEF population estimates were higher in healthy males vs. females (456.8±130.1 vs. 293.9±89.9 liters/minute [mean±SD]). After adjusting for height, estimates remained higher for males (83.1±22.4 vs. 70.9±21.5 liters/m3/minute) and higher for younger (65-79 years) vs. older (≥80 years) participants (78.9±19.7 vs. 62.9±26.6 liters/m3/minute). These results may serve as PEF population reference values for healthy older adults in the US.

